# The TZM-bl Reporter Cell Line Expresses Kynureninase That Can Neutralize 2F5-like Antibodies in the HIV-1 Neutralization Assay

**DOI:** 10.3390/ijms23020641

**Published:** 2022-01-07

**Authors:** Vladimir Morozov, Sylvie Lagaye, Alexey Morozov

**Affiliations:** 1 Institute of Human Virology, University of Maryland, Baltimore, MD 21201, USA; 2 Department of Infectious Diseases, Robert Koch Institute, 13353 Berlin, Germany; 3 Centre de Recherche Saint-Antoine (CRSA), INSERM—UMR-S 938/Sorbonne Université, CEDEX 12, 75571 Paris, France; sylvie.lagaye@inserm.fr; 4 Laboratory of Regulation of Intracellular Proteolysis, Engelhardt Institute of Molecular Biology, Russian Academy of Sciences, 119991 Moscow, Russia

**Keywords:** TZM-bl cell line, HIV-1, mAb 2F5, mAb 4E10, sera, neutralization assay, kynureninase, expression

## Abstract

Induction of broadly neutralizing antibodies targeting ectodomain of the transmembrane (TM) glycoprotein gp41 HIV-1 provides a basis for the development of a universal anti-viral vaccine. The HeLa cell-derived TZM-bl reporter cell line is widely used for the estimation of lentiviruses neutralization by immune sera. The cell line is highly permissive to infection by most strains of HIV, SIV, and SHIV. Here we demonstrated that TZM-bl cells express a 48 kDa non-glycosylated protein (p48) recognized by broadly neutralizing monoclonal antibody (mAb) 2F5 targeting the ELDKWA (aa 669–674) epitope of gp41TM of HIV-1. A significant amount of p48 was found in the cell supernatant. The protein was identified as human kynureninase (KYNU), which has the ELDKWA epitope. The protein is further called “p48 KYNU”. The HIV-1 neutralization by mAb 2F5 and 4E10 in the presence of p48KYNU was tested on Jurkat and TZM-bl cells. It was demonstrated that p48KYNU reduces neutralization by 2F5-like antibodies, but it has almost no effect on mAb 4E10. Therefore, p48KYNU can attenuate HIV-1 neutralization by 2F5-like antibodies and hence create false-negative results. Thus, previously tested immune sera that recognized the ELDKWA-epitope and demonstrated a “weak neutralization” of HIV-1 in TZM-bl assay should be reevaluated.

## 1. Introduction

HeLa cells and HeLa-derived modified cell lines are the most frequent in vitro models that are used for diverse biomedical applications. Compared to the 16 tissues from Illumina Human BodyMap 2.0 in the HeLa Kyoto cell line there are about 1900 highly expressed genes and above 5000 genes that are downregulated or not expressed [1]. Indeed, modifications of the HeLa cells using integrating vectors may result in activation of additional cellular genes and expression of an extra protein(s). Most likely the protein(s) would not interfere with a newly designed cell function. Though, in some instances, the interference might take place.

The TZM-bl reporter cell line (NIH AIDS Reagent program #8129, also called JC57BL-13) was engineered from HeLa cells by amphotropic retroviral transduction to express CD4, CXCR4, and CCR5 [2]. Then cells were transfected with two lentiviral vectors coding for the reporter genes—firefly luciferase [3] and *E. coli* β-galactosidase under control of the HIV-1 LTR [4,5]. After infection of TZM-bl cells, reverse transcription, and integration of viral cDNA, the Tat protein is synthesized from viral transcripts and triggers the expression of introduced reporter genes in cells. The activity of the β-galactosidase may be measured using different assays reflecting the number of cells that turn blue after X-gal staining and the activity of luciferase is quantified by the luminescence intensity that is proportional to the number of infectious viruses present in the examined specimen. Altogether, the cell line is a sensitive and easy diagnostic tool that is widely used to estimate HIV, SIV, and SHIV infectious titer and to evaluate the neutralization (NT) potency of tested sera [6,7,8,9,10,11,12,13,14,15,16,17,18,19,20,21]. It has been shown that TZM-bl cells are infected with ecotropic gamma-retrovirus [22]. However, this contamination does not influence the “reporter ability” of the cell line [23]. Not long ago the protocol of HIV-1 neutralizing antibody testing on TZM-bl cells was optimized and the test was validated by several laboratories [24]. 

Broadly neutralizing monoclonal antibody (mAb) 2F5 recognizes epitope ELDKWA in the membrane proximal external region (MPER) of transmembrane (TM) protein gp41 of HIV-1 [25,26]. MAb 2F5 is frequently used in biochemical, diagnostic, and immunological studies. The antibody was used in passive immunization trials [27,28] and protection studies using adeno-associated virus with inserted genes encoding neutralizing antibodies that demonstrate promising results [29]. An HIV vaccine inducing a high titer of 2F5-like broadly neutralizing antibodies would be a fundamental achievement in the fight against AIDS. However, till now all attempts to design such a vaccine were unsuccessful. 

Here we identified and characterized a protein that is expressed and secreted from the TZM-bl cells and interferes with mAb 2F5 in the HIV-1 neutralization assay.

## 2. Results

### 2.1. Detection and Characterization of p48 from TZM-bl Cells

**Figure 1:**
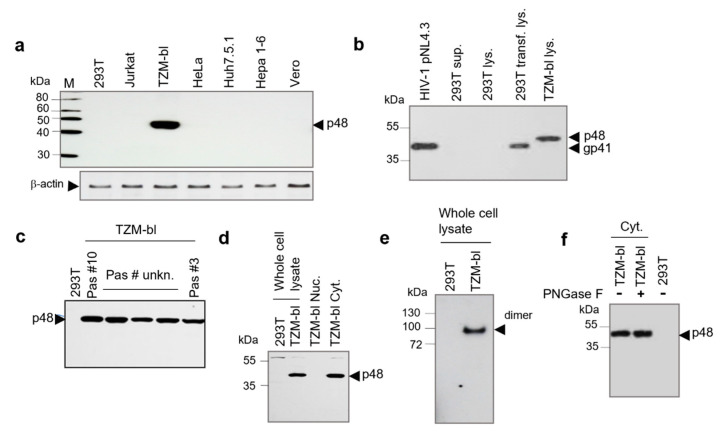
Detection and characterization of the 48 kDa protein (p48) from TZM-bl cells. (**a**) Comparative analyses of established cell lines lysates by Western blotting. Low panel - blot treated with mAb anti-β-actin. (**b**) Comparison of Mab 2F5 reactivity with gp41 HIV-1 and p48 by Western blot analysis. Tracks in order: Pelleted HIV-1_pNL4-3_; Pelleted supernatant (sup.) from HEK293T cells (negative control); Lysate (lys.) of non-transfected HEK293T cells (negative control); Lysate of HEK293T cells transfected (transf.) with HIV-1_pNL4-3_; Lysate of TZM-bl cells. (**c**) Expression of p48 is ubiquitous in TZM-bl cell lines obtained from different sources. Whole-cell lysates of TZM-bl cells passage #10 and #3 following receiving from NIH and lysates from cells obtained from three laboratories (unknown passage numbers following receiving from NIH) were analyzed by Western blot. HEK293T cells whole-cell lysate was used as a negative control. (**d**) Comparative Western blot analysis of nuclear (Nuc.) and cytoplasmic (Cyt.) fractions. The protein was detected in the cytoplasmic fraction. (**e**) Dimerization of p48 was analyzed using native gradient PAGE followed by Western blot analysis using HEK293T and TZM-bl whole cell lysates. The position of the p48 dimer is indicated. (**f**) The p48 protein is not glycosylated as shown by the PNGase F treatment of TZM-bl cytoplasmic fraction (Cyt.). HEK293T whole cell lysate was used as a negative control. Western blots were treated with mAb 2F5 (2.5 mg/mL) and anti-human IgG conjugated with HRP (dilution 1:10 K). M—Size markers (Magic Mark XP standard, Invitrogen) and PageRuler Plus protein ladder (ThermoFisher Scientific, Waltham, MA, USA) were used for calibration.

To study possible non-specific interaction of mAb 2F5 with cellular proteins, lysates of seven cell lines were tested using Western blot analyses. Only in the case of TZM-bl cells, a strong reaction with the 48 kDa protein (p48) was observed (Figure 1a). To examine the consistency of the p48 expression in TZM-bl cells, lysates of cells harvested after passages #10 and #25 were compared by Western blot analysis using mAb 2F5. Both lysates contained equal amounts of proteins (Figure S1). To demonstrate that p48 and gp41TM of HIV-1 are different proteins, a comparative Western blot analysis was performed using HIV-1_pNL4-3_ purified on 20% sucrose cushion, cellular lysates of 293T cells transfected with pNL4-3 (positive control) and cellular lysate of TZM-bl cells. It was shown that the protein mass is different. However, mAb 2F5 reacted with p48 and gp41 equally strong (Figure 1b). Next, we examined the distribution of p48 in five TZM-bl cell lines from three laboratories (Figure 1c, tracks 3 to 5). Cell lines were examined after passage #10 (Figure 1c, track 2) and passage #3 (Figure 1c, track 6) after receiving from the NIH AIDS Reagent Program. The passage number of the other three cell lines originally also obtained from NIH was unknown. All cellular lysates contained p48, indicating that the protein expression in TZM-bl cells is ubiquitous. Afterwards, we analyzed the p48 localization in cells. Extracted cytoplasmic and nuclear fractions of TZM-bl cells were tested by Western blot analysis with mAb 2F5. The p48 was detected only in the cytoplasmic fraction (Figure 1d, track 4). Further on, oligomerization of p48 was assessed using a cytoplasmic fraction of TZM-bl cells. Protein electrophoresis was performed in native 4–20% gradient gel. Performed Western blot analyses demonstrated that the protein is forming dimers (Figure 1e, track 2). 

To access the protein glycosylation, the cellular lysate was treated with peptide-N-Glycosidase (PNGase F). As shown by Western blot analysis mobility of the protein was not changed, indicating a lack of *N*-glycosylation (Figure 1f). Altogether, performed tests showed that p48 is a cytoplasmic, non-glycosylated protein that in native conditions forms homodimers. It should be emphasized, that no immune reactivity against p48 was detected by Western blot analysis when TZM-bl cellular lysates were examined using mAb 4E10 and Chessie 8 (Figure S2), that target other than 2F5 regions of gp41 HIV-1 

### 2.2. Analysis of the p48 in Cells by Confocal Microscopy and Isolation of p48 from Cell-Free Supernatant

**Figure 2:**
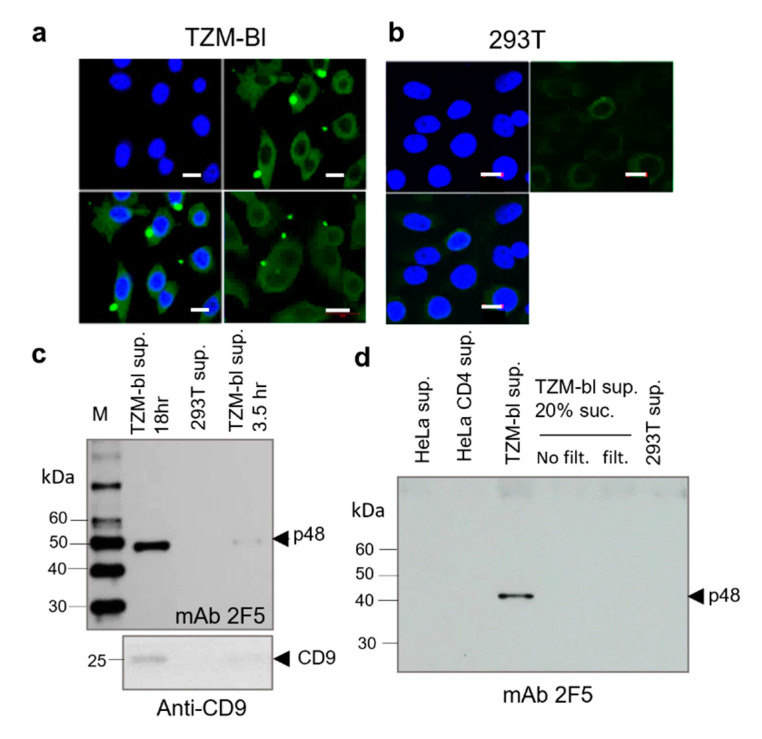
Confocal microscopy of TZM-bl cells revealed blebs and bright extracellular vesicles containing p48. Isolation of p48 from the cell supernatant. Confocal microscopy. The TZM-bl (**a**) and HEK293T (**b**) cells were fixed, stained with mAb 2F5 (5 µg/mL), and treated with anti-human Alexa 488 conjugated antibodies. Cell nuclei were stained with DAPI. Scale bar = 10 µm. Green blebs and extracellular vesicles were found in association with TZM-bl cells. (**c**) Detection of p48 in the supernatant of TZM-bl cells grown on SFM. Pelleted serum-free supernatant from TZM-bl cells after high-speed centrifugation (170,000× *g*) for 18 hr and 3.5 hr and pellet from the supernatant of HEK293T cells after 18 h of centrifugation were analyzed by Western blot. (Low panel) Detection of CD9 in pelleted supernatants. Blot was treated with anti-human CD9 mAbs in dilution 1:750. (**d**) p48 released also as a free protein. Cell supernatants were centrifuged at 1500× *g*, at 10,000× *g*, and overnight at 170,000× *g*. Tracks in order: Supernatant (sup.) from HeLa cells (overnight centrifugation). Supernatant from HeLa CD4+LTR-Luc cells (HeLa CD4) overnight centrifugation; Supernatant from TZM-bl cells (overnight centrifugation); Supernatant from TZM-bl cells (no 0.45 μm filtration (no filt.), overnight centrifugation through 20% sucrose (suc.)); Supernatant from TZM-bl cells (0.45 µm filtration (filt.) and overnight centrifugation through 20% sucrose); Supernatant from 293T cells (overnight centrifugation). Western blot analyses were performed using mAb 2F5.

The p48 localization in TZM-bl cells was accessed by confocal microscopy (Figure 2a). The TZM-bl cells were fixed and stained with mAb 2F5 (5 µg/mL), followed by treatment with anti-human Alexa 488 conjugated antibodies. The protein was found in the cytoplasm with no evident association with the plasma membrane. Interestingly a high concentration of p48 was found in blebs and extracellular vesicles of various sizes (~0.4–1 µm) (Figure 2a). Such vesicles were not detected in HEK293T cells (Figure 2b). The sizes of vesicles correspond to that of shedding microvesicles (MVs) that are ranging from 100 nm to 1000 nm in diameter. While exosomes are generally smaller (<100 nm) [30].

Observed blebs and extracellular vesicles (Figure 2a) indicated that p48 can be released from cells as a cargo of presumed MVs. In this regard, we attempt to isolate p48 from cell supernatant using extended ultracentrifugation. A significant amount of p48 was detected in pelleted cell supernatant after 18 h of centrifugation. Only traces of p48 were detected after 3.5 h of centrifugation. In both experiments, centrifugation was performed at 170,000× *g* (Figure 2c, upper panel). The simultaneous presence of p48 and CD9 (Figure 2c, low panel) in pellets after centrifugation might be considered as an argument in favor of MVs implication in p48 capture. However, the release of free p48 might also take place, as after ultra-centrifugation through the 20% sucrose cushion the protein content in the pallet was undetectable (Figure 2d) or drastically reduced.

### 2.3. Identification of p48 as Kynureninase (KYNU)

The HXB2 molecular clone was used as a packaging construct in the design of the TZM-bl cell line. Lentiviral transfer vectors coding for the viral receptors and containing 234 nucleotide long RRE sequences (which overlap with gp41) were used in cell modification [2,4,5]. In this regard, we initially suggested that p48 might be coded by one of the mutated lentiviral transfer vectors that contained a fragment of the HIV-1 *env* gene. To detect HIV-1 *env* transcripts the RNA was extracted from TZM-bl and HEK293T cells (negative control) and tested by RT-PCR using two sets of primers (Table 1) targeting the 3′-end of gp120 and the gp41 ectodomain fragments. However, all attempts to detect the *env* transcripts have failed. Altogether, the absence of the *env*-like transcripts and lack of reactivity with human mAb 4E10 and mouse mAb Chessie 8, allowed suggesting, that p48 is a cellular protein. Using bioinformatics, the gp41 HIV-1 ectodomain was examined for homology to cellular proteins and the only protein that has the ELDKWA epitope was human kynureninase (KYNU).

**Figure 3:**
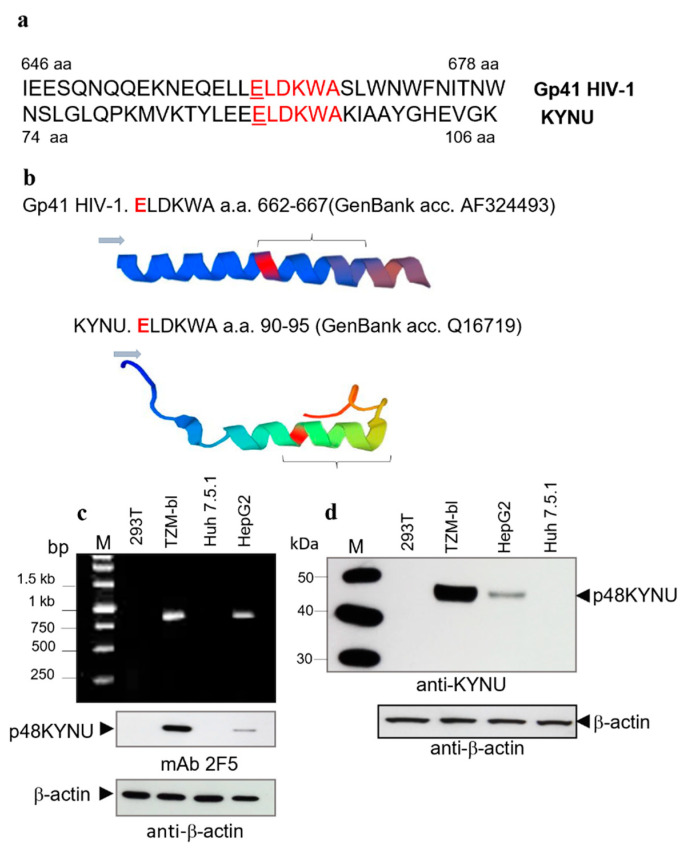
Identification of p48 as kynureninase (KYNU). Detection of p48 KYNU transcripts in TZM-bl cells and comparative analysis of KYNU protein content in TZM-bl and HepG2 cells. (**a**) Amino acid alignment of gp41 HIV-1 and KYNU fragments containing ELDKWA with flanking regions. (**b**) 3D models of gp41 HIV-1 and KYNU (a part of the H4 domain) amino acid fragments containing the ELDKWA epitopes. Blue arrowhead indicates the direction of reading. Braces marked the position of the ELDKWA motifs. Glutamic acids (E) in the ELDKWA epitopes are marked in red. (**c**) Upper panel. Detection of KYNU transcripts in different cell lines by RT-PCR. M—Markers (1 kb O’Gene Ruler). Low panel. Comparative analysis of KYNU in TZM-bl and HepG2 cellular lysates (4 × 10^4^ cells) by Western blot analysis. Samples are in the same order as on the upper panel. Equal protein load was confirmed by anti-β-actin staining. (**d**) Recognition of KYNU in both the TZM-bl cells and the HepG2 cells by anti-KYNU serum. M—Markers (MagicMark XP Standard). Upper panel. Western blot analysis. HEK293T cell lysate (1 × 10^5^ cells); TZM-bl cell lysate (1 × 10^5^ cells); HepG2 cell lysate (1 × 10^5^ cells); Huh 7.5.1 cell lysate (1 × 10^5^ cells). Low panel. Equal protein load was confirmed by β-actin distribution. Western blot analyses were performed using mAb 2F5 (2.5 mg/mL), rabbit anti-KYNU (1:750), and anti-β-actin (1:1000).

KYNU is a key enzyme on the kynurenine pathway of tryptophan metabolism. KYNU is a cytoplasmic, non-glycosylated protein of about 52 kDa (48–50 kDa in SDS-PAGE), that forms homodimers [31,32]. Accordingly, the parameters of p48 were like that of KYNU. Data from Human Protein Atlas (www.proteinallas.org, accessed on 15 March 2018) indicated that several cell lines including human hepatocellular carcinoma cell line HepG2, express KYNU. The crystal structure of KYNU is known [33]. It was found that both proteins contain the ELDKWA epitope. In KYNU the motive is in the H4 domain [34]. Alignments of amino acid fragments with ELDKWA epitopes of gp41 HIV-1 and KYNU (Figure 3a), and 3D models of the regions together with flanking amino acids are shown (Figure 3b). Interestingly, besides the ELDKWA epitopes, these proteins structurally are significantly different.

Next, we performed comparative analyzes of KYNU transcripts in TZM-bl and HepG2 cells. Transcripts from HEK293T and Huh 7.5.1 cells were used as negative controls. To analyze transcripts of p48 KYNU the RT-PCR targeted the 845 bp long fragments with ELDKWA was performed. Corresponding amplicons were detected in both the TZM-bl and the HepG2 cells. The number of amplicons in TZM-bl cells was about 3–4 times higher compared to that of HepG2 (Figure 3c, upper panel). Analyses of sequenced clones demonstrated that KYNU fragments from TZM-bl cells and HepG2 cells were identical and showed 100% identity to human KYNU sequences from the GenBank (NM_001032998, BC000879, NP_001028170.1, NM_003937, Q16719, etc.).

After that, the p48 KYNU protein expression in TZM-bl and KYNU in HepG2 cells were compared by Western blot analysis using mAb 2F5 (Figure 3c, low panel) and polyclonal anti-KYNU serum (Figure 3d, upper panel). The protein was detected in both cell lines. However, the amount of p48 KYNU in TZM-bl was about 8 times higher (estimated using ImageJ) than in HepG2 cells, while the total protein load was equal (Figure 3d, low panel). A significantly higher protein expression in TZM-bl cells might be attributed to the promotion of the KYNU gene expression by LTR from the integrated lentiviral vector. Noteworthy, comparison of polyclonal anti-KYNU serum and mAb 2F5 demonstrated equal reactivity with recombinant (rKYNU) and p48 KYNU when rabbit anti-KYNU was used in dilution (1:800) and mAb 2F5 were diluted to 2.5 mg/mL (Figure S3a,b). 

Lastly, the cDNA KYNU amplicon (845 bp) obtained from TZM-bl cells with a coding capacity of 36 kDa was cloned in the expression vector pTargeT (Promega). HEK293T cells were transfected with the construct and 48 hr later cellular lysates were prepared and examined for KYNU expression by Western blot analysis with anti-KYNU antibodies. The truncated variant - p36 KYNU, was recognized by anti-KYNU (Figure S4). The protein was used as a target antigen in several experiments (not shown).

Thus, obtained results explicitly demonstrated that the p48 protein in TZM-bl cells is a human kynureninase.

### 2.4. Estimation of p48 KYNU Amount in TZM-bl Cells and Cell Supernatant

Estimation of p48 KYNU content in cells and especially the amount of the protein released from cells were critical for the prediction of p48 KYNU potency to neutralize 2F5-like antibodies.

**Figure 4:**
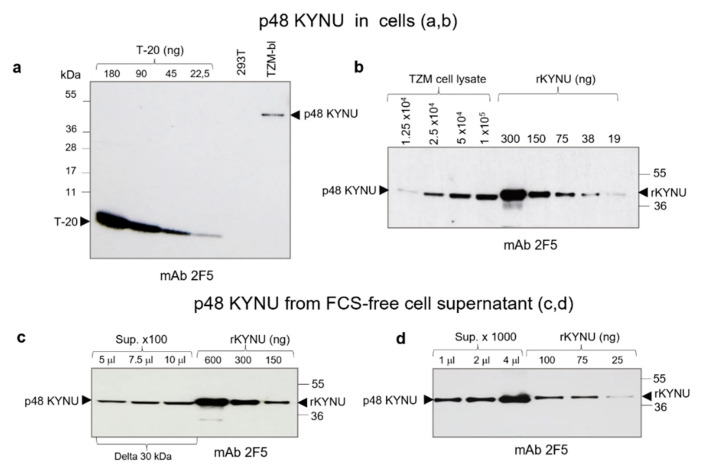
Estimation of p48 KYNU content in cells (a, b) and in cell supernatant (c, d). (**a**) Quantification of p48 KYNU in lysate from 5 × 10^4^ TZM-bl cells was performed using a serial dilution of the T-20 peptide that contains the ELDKWA epitope. HEK293T cellular lysate from 5 × 10^4^ cells was used as the negative control. It was estimated that approximately 32 ng of p48 KYNU were present in 5 × 10^4^ cells. That amount of p48 KYNU corresponds to ~1.6 × 10^7^ molecules/cell. Western blot analysis was performed using mAb 2F5. (**b**) Quantification of p48 KYNU using the rKYNU as a reference. Serial two-fold dilutions of cellular lysates from 1 × 10^5^ to 1.25 × 10^4^ cells and serial two-fold dilutions of rKYNU from 300 ng to 19 ng (shown above) were examined. Using the ImageJ software, it was shown that the amount of p48 KYNU in 1 × 10^5^ cells was close to that of 130 ng of rKYNU (~1.6 × 10^7^ molecules/cell). (**c**) Cell-free supernatant from TZM-bl cells harvested after 48 h and concentrated 100 times using 30 kDa molecular weight cut-off concentrators (Sartorius, Stedim, Germany). Tracks: samples load 5 µL (equivalent of 0.250 mL); 7.5 µL (equivalent of 0.750 mL); 10 µL (equivalent of 1 mL); serial dilutions of rKYNU (amounts in ng are given below). (**d**) Estimation of p48 KYNU amount in pelleted FCS-free supernatant of TZM-bl cells concentrated 1000 times. Two-fold dilution of concentrated cell supernatant (concentration ×1000) from TZM-bl cells and serial dilutions of recombinant rKYNU were tested. Tracks: samples content 1 µL (equivalent of 1 mL of pelleted supernatant); 2 µL (equivalent of 2 mL); 4 µL (equivalent of 4 mL); 100 ng of rKYNU, 75 ng; 50 ng; 25 ng. Using ImageJ, it was shown that 1 µL contains ~1.2 × 10^9^ molecules of p48 KYNU. Blot was treated with mAb 2F5 (2.5 mg/mL).

Initially, we estimated the amount of p48 KYNU in TZM-bl cells. Cells were grown in complete medium and were harvested after 48 h. The p48 KYNU content in lysate from 5 × 10^4^ TZM-bl cells was compared to serial two-fold dilutions (from 180 ng to 22.5 ng) of T-20 (a 36 aa peptide with ELDKWA epitope) (Figure 4a). Comparative analysis using ImageJ showed that lysate of 5 × 10^4^ cells contained about 32 ng (or 4 × 10^11^ molecules) of p48 KYNU. That amount corresponds to 0.64 pg or 0.8 × 10^7^ molecules/cell. Evidently, this amount is lower than the amount of p48 KYNU in cells, as a part of the molecules might be captured and pelleted together with cellular debris during centrifugation.

Then, we performed titration of TZM-bl cell lysates using recombinant KYNU (rKYNU) as a reference (Figure 4b). Two-fold serial dilutions of TZM-bl lysates from 1.25 × 10^4^ to 1 × 10^5^ cells were compared with serial dilutions of rKYNU (from 300 ng to 19 ng) (Figure 4b). Analysis of bands intensity was performed using the ImageJ software. The amount of p48 KYNU in the lysate from 1 × 10^5^ cells corresponded to 130 ng or 1.6 × 10^12^ molecules of rKYNU. Dividing by the cell number it comes with 1.6 × 10^7^ molecules/cell. So, likely because of the difference in references used and preparations the amount of p48 KYNU varies from 0.8 to 1.6 × 10^7^ molecules/cell with a mean value of 1.2 × 10^7^ molecules/cell. It is worth mentioning, that rKYNU used in this study has a 6-His tag, which increases the size of recombinant protein by 0.93 kDa.

Next, we estimated the p48 KYNU content in FCS-free supernatant from TZM-bl cells. Ten milliliters of medium from 8 × 10^6^ TZM-bl cells were harvested after 48 h and processed by low-speed centrifugation at 1200 *g* and 10,000 *g* for 10 min. The supernatant was concentrated 100 times using a 30 kDa molecular weight cut-off concentrator (Sartorius, Stedim, Göttingen, Germany). A serial two-fold dilution of p48 KYNU from concentrated supernatant and rKYNU (used as reference) were compared by Western blotting (Figure 4c). Estimation of the p48 KYNU content was accessed using ImageJ software. It was shown that 750 μL of supernatant contained 150 ng of p48 KYNU (or 0.2 ng/μL). As one mL of cell medium contained KYNU that was released from 8 × 10^5^ cells (1/10 of all cells). Dividing 150 ng by 8 × 10^5^ (cells) results in 0.187 pg/cell. Using this calculation, we estimated the putative release of p48 KYNU from one well of the 96 well plate. Counting 4 × 10^4^–5 × 10^4^ cells at the confluence, after 48 h from 7.5 ng to 9.35 ng of p48 KYNU might be released.

If counting interaction of p48 KYNU with IgG recognizing the ELDKWA epitope as 1:1 and considering that 1 ng of IgG = 4.3 × 10^9^ molecules and 1 ng of p48 KYNU = 1.2 × 10^10^ (or 0.6 × 10^10^, if 1:2) molecules, that would result in a 2.8:1 (2.8 ng to 1 ng) ratio called further the “interaction factor” (“inf”) of the IgG—p48 KYNU pair. If considering the “inf”, 7.5 ng to 9.35 ng of p48 KYNU can interact with 21–26 ng of 2F5-like antibodies in a single well of the 96 well plate. It should be noted that cells are permanently producing p48 KYNU, thus the implication of the half-life parameter might not be so critical.

P48 KYNU was also isolated from FCS-free supernatant using ultracentrifugation. As above, TZM-bl cells were grown for 48 h, 10 mL of medium was processed, and the supernatant was centrifuged overnight at 170,000 *g*. The precipitate was recovered in PBS to achieve 1000 times concentrate (Figure 4d). As estimated by Western blotting, the concentration of p48 KYNU was from 125 ng/mL (0.156 pg/cell) to 150 ng/mL (0.187 pg/cell) confirming the above-described estimations of extracellular p48 KYNU concentration. The procedure of protein isolation from SFM was repeated three times and the amounts of recovered protein varied insignificantly.

When using the pellet from SFM that was concentrated 1000 times the total protein load was ~1 µg/µL, while when using complete DMEM, it was about 10 times higher. Thus, p48 KYNU from SFM was selected for testing in the virus-neutralization (NT) assay.

### 2.5. Evaluation of the p48 KYNU Effect on mAbs 2F5 and 4E10 in NT Assays

**Figure 5:**
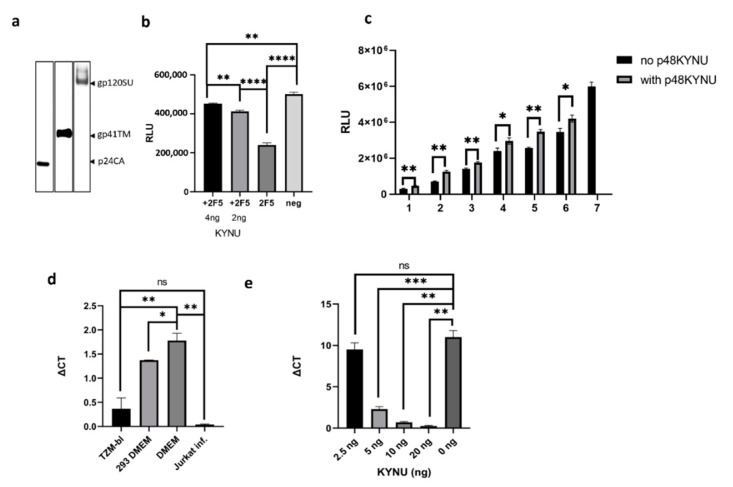
Neutralization of Mab 2F5 by p48 KYNU in HIV-1 NT assay. (**a**) HIV-1 preparation used in NT assays. Analysis of HIV-1 integrity by Western blotting (detection of p24 CA, gp41 TM, and gp120 SU). A Western blot analyses were performed using goat anti-p24CA (1:1000), goat anti-gp120 (1:1000) and Mab 2F5 (2.5 mg/mL). (**b**) NT assay on TZM-bl cells. Amounts of p48 KYNU added to the reaction mixture are shown below the columns. 100 ng of mAb 2F5 were added to two reaction mixtures. 2F5+KYNU—Mixture containing mAb 2F5 and 4 or 2 ng of extra p48 KYNU (indicated as +2F5). 2F5 mixture without additional p48 KYNU (indicates as 2F5). Neg.—Negative control, mixture without 2F5 and KYNU. RLU—Relative luciferase units. (**c**) NT assay on TZM-bl cells. 1 to 6—Serial two-fold dilution of the reaction mixture that initially contained 100 ng of MAb 2F5 and 5 ng of p48 KYNU. 7—Reaction mixture without MAb 2F5 and p48 KYNU. Black columns—Mixtures without p48 KYNU, grey columns—mixtures containing p48 KYNU. The virus was taken to achieve MOI 0.5. (**d**) NT assay on Jurkat cells. 100 µL of SFM from TZM-bl that contained app. 10 ng of p48 KYNU was mixed with mAb 2F5 (100 ng/reaction). Each well contained 5 × 10^5^ cells. Each culture medium was diluted 1:1 with complete RPMI 1640. A complete fresh DMEM medium was used as a negative control. The virus was added to achieve MOI of 0.5. The DNA was extracted from cells after 36 h. TZM-bl—supernatant from TZM-bl cells (contained 10 ng of KYNU), 293 DMEM—Cell-free supernatant from HEK293T cells, DMEM—Fresh complete DMEM with 10% FCS; DNA from HIV-1 infected Jurkat cells was used as a positive control. Quantification of integrated proviruses was performed by real-time PCR. GAPDH was used as a housekeeping gene. Delta C_t_ = C_t_ target—C_t_ GAPDH. (**e**) NT assay on Jurkat cells using mAb 2F5 (100 ng/reaction) and increasing concentrations (from 2.5 ng to 20 ng) of rKYNU. Amounts (ng) of applied rKYNU are given below the columns. Quantification of integrated proviruses was performed by real-time qPCR. GAPDH was used as a housekeeping gene. The virus infectious titer was 5 × 10^5^. Experiments were performed in triplicates. Error bars represent standard deviation. An unpaired t-test was used to evaluate the statistical significance. The *p* values less than 0.05 were regarded as statistically significant. Asterisks indicate: * *p* < 0.05; ** *p* < 0.01; *** *p* < 0.001; **** *p* < 0.0001. *p*-value calculations were performed using GraphPad Prism version 8.4.3. (GraphPad Software, San Diego, CA, USA; RRID: SCR_002798) software.

In the absence of a reference serum with a known proportion of the ELDKWA epitope recognizing 2F5-like neutralizing antibodies, precise estimation of p48 KYNU interference with corresponding antibodies and the impact on virus neutralization was difficult to achieve. In part, the problem was solved by mixing the mAbs 2F5 (or 4E10) with normal human serum. These mixtures were used to monitor the effect of p48 KYNU in the NT assay. NT assays were performed using HIV-1_pNL4-3_ as a target (Figure 5a) and it was performed on TZM-bl and Jurkat cells.

When the NT assay was performed on TZM-bl cells, an extra amount (4 ng or 2 ng/well) of p48 KYNU isolated from pelleted supernatant was added to the reaction mixture. The reason to use additional p48 KYNU was to better approximate the protein effect on tested sera. In this assay, the initial concentration of mAb 2F5 was 100 ng/well (Figure 5b). Since 7.5–9.35 ng (mean value 8.5 ng) of KYNU is released from one well of 96 well plate contains during 48 h, together with applied protein, that would result in a maximum of 12.5 ng (8.5 ng + 4 ng) of p48 KYNU in a single well. Considering the “inf” factor, that amount of KYNU can potentially interfere with up to 35 ng or 35% of used antibodies. The obtained results were congruent with the predicted values although demonstrated even higher interference and indicated 80% and 65% 2F5 neutralization reduction following the supplementation of the reaction mixture with 4 or 2 ng of p48 KYNU, respectively (Figure 5b). In a parallel setting, a control dilution of mAb 2F5 without p48 KYNU was performed. When mAb 4E10 were used in the NT assay together with p48 KYNU much less reduction of neutralization was observed (Figure S5).

Another NT test on TZM -bl cells was performed using a serial two-fold dilution of mAb 2F5 with and without p48 KYNU. Initially, the concentration of mAb 2F5 was 100 ng with 5 ng of p48 KYNU (Figure 5c).

The NT assay on Jurkat cells (KYNU free cells) was performed to estimate the interaction of mAb 2F5 with native p48 KYNU from the supernatant of TZM-bl cells. The efficacy of virus neutralization was assessed by quantifying integrated HIV-1 proviruses. Analysis of DNA by RT- PCR demonstrated that the supernatant containing p48 KYNU increased the number of integrated proviruses by more than one Ct (ΔC_t_~1) when compared to supernatant from HEK293T cells (Figure 5d). Thus, 10 ng of p48 KYNU can reduce virus neutralization of mAb 2F5 (50 ng) by approximately two times. While the impact of supernatant from HEK293T cells on HIV-1 neutralization was insignificant.

Another NT assay was performed on Jurkat cells using commercial rKYNU. The goal of the assay was to estimate the amount of rKYNU that can inactivate 50 ng of mAb 2F5 in one well of 96 well plate. To achieve this goal, an increasing concentration of rKYNU from 2.5 ng/well to 20 ng/well were added to reaction mixtures (Figure 5e) and, 48 h later, the DNA was extracted and analyzed for the proviruses as described above. In this case, the inactivation of mAb 2F5 by rKYNU was prominent. For instance, when 5 ng of rKYNU were used, the neutralization was reduced more than four times (ΔC_t_~2), while 20 ng of the rKYNU reduced neutralization by more than 1000 times (ΔC_t_~10). The stronger effect of rKYNU in NT assay on Jurkat cells was most likely attributed to the higher purity of rKYNU that does not contain cellular debris.

## 3. Discussion

The design of an efficient anti-HIV vaccine remains a principal, but a still inaccessible goal. The main hurdles on this way are—virus genetic variability and a dense glycan shield that includes ~25 glycans in SU and 7 in TM (4 glycans are in the ectodomain). Numerous approaches were used to obtain broadly neutralizing antibodies targeted the MPER of gp41TM and, in particular, the ELDKWA epitope—one of the most conserved regions. 

Success in vaccine development depends not only on the selection of the right immunogen. Other important factors are the specificity and sensitivity of the reference systems, which are used for testing virus neutralization. One of the systems is the HeLa-derived TZM-bl reporter cell line, which is one of the most frequently used systems, that allow to quantify HIV infectious titer and to analyze the neutralization potential of anti-viral immune sera. 

Here, we demonstrated that a TZM-bl cell expresses a protein (p48) that interferes with immune sera during HIV-1 neutralization assays. Based on biochemical parameters [32], immunoreactivity, and sequence, the protein was identified as *L*-kynurenine hydrolase (KYNU). Previously, using the immunoprecipitation approach and protein array studies, it has been shown, that mAb 2F5 recognizes KYNU via its ELDKWA epitope [34]. It should be emphasized that mAb 2F5 is highly specific and does not react with mutated ELD/EKWA epitope as in KYNU of opossum [34]. The implication of KYNU in reduced frequency of 2F5-like neutralizing antibodies in HIV-1 infected humans was also discussed [34]. 

KYNU is a member of the aspartate aminotransferase superfamily. It catalyzes the cleavage of kynurenine into anthranilic acid in the kynurenine pathway of tryptophan metabolism [31]. It is a plasma protein that has the highest level of expression in the liver [35]. To a significantly less degree, KYNU is expressed in the heart, brain, placenta, lung, liver, skeletal muscle, kidney, and pancreas. Interestingly, pro-inflammatory cytokines like IFN-γ and TNF-α can notably increase KYNU expression in human fibroblasts [36]. KYNU is highly upregulated in patients with chronic inflammatory skin disease and tryptophan metabolites downstream of KYNU induce inflammatory gene expression [37]. 

Besides the TZM-bl cells, other cell lines are expressing KYNU (Human Protein Atlas; www.proteinatlas.org, accessed on 13 March 2016). Two of them U87MG (ATCC HTB-14, human glioblastoma epithelial cells) and A549 (ATCC CCL-185, human alveolar adenocarcinoma epithelial cells) demonstrated a significant level of KYNU expression with 309 and 169 transcripts per kilobase million (TPM), respectively. The level of KYNU expression in HeLa and HepG2 cells is lower—139 and 88 TPM, accordingly. A diverse level of KYNU expression in cells might be a result of aneuploidy and/or aberrations during alternative splicing of the transcript. It is likely, that KYNU expression in HeLa cells is not a property of all the HeLa strains. As a result of 70 years of cultivation, the HeLa cell proteome can vary from one lab to another [38]. Lysates from three strains of HeLa were examined by Western blotting; only HeLa CD4+LTR-Luc cell line expressed KYNU. The HeLa-CD4 strain HI-J was used for the TZM-bl cell design. Unfortunately, in the Human Protein Atlas, the parental HeLa strain that has been tested for the KYNU transcripts was not mentioned. 

Identification of the p48 KYNU in the supernatant of TZM-bl cells was unanticipated and raised two questions. First, how the protein from cytoplasm is released from cells in the absence of the exportation signal and the transmembrane domain (showed using the Octopus topology program, http://octopus.cbr.su.se, accessed on 08 July 2016). Second, how important might be p48 KYNU interference with anti-ELDKWA immune sera in NT assay?

To access the first question, we suggested that the release of p48 KYNU might occur in a non-conventional way, which utilizes the intracellular vesicles secretion route. Such a release was proposed for the interleukin-1β (IL-1β) [39]. It is also known, that shedding MVs are extracellular transporters for the cellular cargo and can deliver leaderless proteins [40]. This type of secretion was demonstrated for the IL-1α, fibroblast growth factor [41], and many other proteins, peptides, nucleic acids, and fragments of viruses.

To gain proves of possible non-classical secretion of KYNU we performed in silico analyzes using the SecretomeP2 program. The probability of non-classical secretion was estimated as 0.47 and that was close to the threshold level of 0.5. However, when the N-terminal fragments of the protein were examined an 11 amino acid (residues C21 to H31) long fragment at the N-terminal part of p48 KYNU demonstrated a 0.75 probability of non-classical secretion. Whether this stretch is a key element required for secretion remains to be determined. Moreover, it was found that filtration of TZM-bl FCS-free supernatant through the 0.45 µm and 0.22 µm filter and 20% sucrose cushion during ultra-centrifugation drastically reduced the amount of pelleted p48 KYNU (Figure 2c). However, if omitting 20% sucrose cushion and filtration, and performing overnight centrifugation, the amount of p48 KYNU increased significantly.

Taken together the results of confocal microscopy and centrifugation indicate that likely p48 KYNU is released from cells by two ways, as a free protein and in vesicles (presumably MVs) as a cargo. Otherwise, it can be efficiently liberated from vesicles following secretion.

The second question to answer was: “To what extent does the p48 KYNU inhibit sera, which contain 2F5-like neutralizing antibodies?

There was a considerable variability of gp41-derived immunogens and delivery strategies that were proposed to boost the 2F5-like neutralizing antibodies [42]. In terms of TZM-bl assay, it is likely that dependent on the antigen that has been used; the effect of KYNU on immune sera might vary significantly.

The HIV-1 gp41 is a strong immunogen that has six major antigenic determinants in the ectodomain [43,44,45,46]. Determinant I (residues 597-613), Determinant II, III, IV—(residues 641–683), Determinant V—(residues 512-579), Determinant VI—(residues 611–640) [44]. However, Determinant III in general and, the ELDKWA epitope in particular, are poorly exposed on the surface of the virion and are not immunodominant [44,47,48]. That is one of the reasons why neutralizing antibodies targeting the ELDKWA epitope in HIV-1 infected humans are scarce [49]. Such antibodies might appear only 20–30 months after virus infection [50]. However, delay with antibodies appearance may be explained not only by extended antibody maturation, because of a complex CDRH3 loop structure [51], but considering the obtained results, also by the time that is required to break natural tolerance against KYNU [34]. 

So, what type of immune serum might be predominantly affected by p48 KYNU in TZM-bl NT assay? For simplicity let’s compare two hypothetical immune sera rose against different immunogens but recognizing the ELDKWA epitope. For example, the first group of sera was induced by immunization with the entire gp41TM or the gp41 ectodomain. The antigen may be administered in different formulations such as virus-like particles, liposomes, chimeric proteins, recombinant proteins, etc. In this case, the anti-MPER, including ELDKWA, immune responses might be relatively weak, because the domain is poorly immunogenic. However, the chance to obtain neutralizing antibodies recognizing required pre-hairpin conformation of gp41 [52,53,54] might not be zero. The second group of sera was obtained after immunization with peptide(s), that span the entire MPER (aa 662–683, acc. AF324493), or shorter peptides, containing the ELDKWA epitope as a part. In this case, the immune response might be directed predominantly against Determinant III including the ELDKWA epitope. Therefore, the overall immune response against this epitope might be relatively strong [55,56,57] (Morozov VA, personal communication), but antibodies are not neutralizing. Thus, if using the immunogens of the second type, the effect of KYNU might be low, as an outcome of a high amount of binding, but not neutralizing, antibodies. Nevertheless, compared to the first group of immunogens, because of the conformation, the chances to obtain neutralization antibodies using peptides are significantly lower, if any [16]. Therefore, we suggested that immune serum derived against the entire gp41 (or its large fragment) that contains a relatively low amount of anti-ELDKWA antibodies (recognizing and neutralizing) might be mostly “neutralized” by KYNU. It should be emphasized, that recognizing antibodies may also contribute to neutralization [58]. 

Valuation of KYNU interaction with immune serum-containing both recognizing and 2F5-like neutralizing antibodies might be more sophisticated. First, the interaction depends on the proportion of the 2F5-like neutralizing antibodies in a pool of antibodies recognizing the ELDKWA epitope. Second, the condition of serum, including the half-life of both tested immunoglobulins and KYNU should be considered. The last parameter is likely less critical, as during the assay period the p48 KYNU is permanently released from cells.

**Figure 6:**
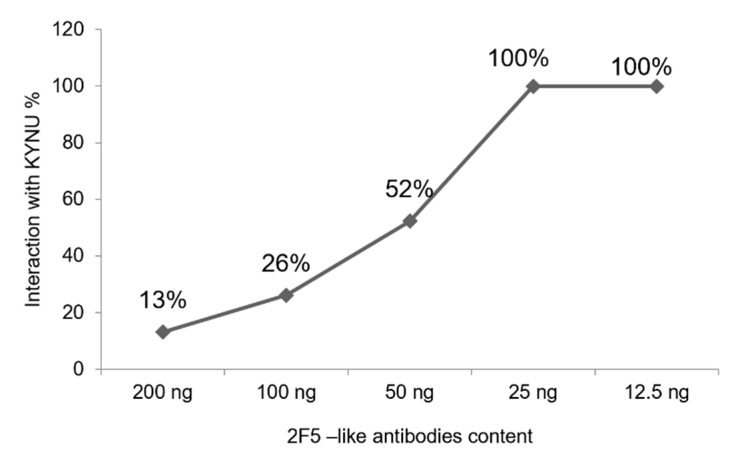
Estimation of p48 KYNU interaction with different amounts of 2F5-like antibodies. Estimation is based on the KYNU amount detected in the supernatant of TZM-bl cells (up to 9.35 ng) and the interference factor of 2.8. Here we consider only strong interactions between 2F5-like antibodies and p48 KYNU.

Collectively our data indicate that the amount of KYNU in a single well of the 96 well plate may be sufficient to neutralize approximately 26 ng of 2F5-like neutralizing antibodies. Based on these values we obtained a graph displaying predicted interference of KYNU with the neutralization potency of sera (Figure 6).

Thus, if the aliquot of tested antibodies contains less than 26 ng of ELDKWA-binding 2F5-like antibodies theoretically they all might be neutralized by KYNU. However, during NT assay a weak interaction of recognizing (not neutralizing) antibodies can reduce interaction of KYNU with neutralizing 2F5-like antibodies from the pool. 

In this regard previously detected “weak neutralizing” sera (antibodies) should be retested using other detection systems. 

## 4. Materials and Methods

### 4.1. Cells and Cellular Lysates

The established cell lines used in this study are listed in Table 1.

TZM-bl cell line was obtained through the NIH AIDS Reagent Program, Division of AIDS, NIAID, NIH from Dr. John C. Kappes, Dr. Xiaoyun Wu, and Tranzyme Inc. (Durham, NC, USA). Adherent cells were maintained in DMEM and lymphoid cells in RPMI 1640 (Jurkat) supplemented with 10% heat-inactivated fetal bovine serum (FBS), 1% penicillin-streptomycin, and L-glutamine. TZM-bl cells were also grown on serum-free medium (SFM) using as a substitution LiforCell (Lifeblood medical, Inc., Adelphia, NJ, USA) and XerumFree (TNCBio, Eindhoven, The Netherlands) mediums supplemented with antibiotics and L-glutamine, as described above. Cell lines were tested for mycoplasma contamination using the MycoSensor PCR assay kit (Agilent Technologies, Santa Clara, CA, USA) and were found mycoplasma free.

To prepare cellular lysates cells were washed with phosphate-buffered saline (PBS), harvested and three times centrifuged at 1200 g for 5 min. Obtained pellets were treated with NP40 lysis buffer (1% NP-40; 150 mM NaCl; 50 mM Tris-HCl pH 8.0) containing protease inhibitor cocktail Complete (Roche, Mannheim, Germany). After incubation on ice for 10 min, the lysates were centrifuged at 10,000 g for 10 min. Supernatants were harvested and were kept frozen at −80 °C before use. Stepwise separation of nuclear and cytoplasmic extracts from TZM-bl cells was performed according to the protocol of the Nuclear and Cytoplasmic Extraction Reagents kit (Thermo Fisher Scientific Inc., Waltham, MA, USA).

### 4.2. Monoclonal Antibodies (mAb), Sera, Peptide, and Recombinant Protein

Human mAbs 2F5 (core epitope ELDKWA) and 4E10 (core epitope NWFDIT) that targeted the membrane proximal external region (MPER) of gp41TM HIV-1 were from Polymun Scientific (Vienna, Austria). Mouse anti-gp41 monoclonal antibodies Chessie 8 (core epitope PDRPEG) was obtained from NIH AIDS reagent Program (Catalog # 526) and also was kindly provided by Dr. George Lewis (Institute of Human Virology, University of Maryland, Baltimore, MD, USA). Rabbit anti-human kynureninase serum was obtained from GeneTex Inc. (Irvine, CA, USA). Monoclonal anti-β-actin clone AC-74 was from Sigma (St. Louis, MI, USA). Anti-human and anti-rabbit IgG- horse reddish peroxidase (HRP) conjugates were obtained from Dako Laboratories (Glostrup, Denmark). Baculovirus-derived recombinant KYNU (rKYNU) was purchased from R&D Systems Inc. (Abingdon, UK). Mouse mAb anti-CD9 antibody was obtained from Invitrogen (Carlsbad, CA, USA). T-20 (Enfuvirtide, Fuzeon^®^) a synthetic 36-residues peptide (4.4 kDa) corresponding to the amino acid residues 127-162 (GenBank acc. AF324493) of the heptad repeat 2 (including the ELDEKWA epitope) of gp41 HIV-1 was kindly provided by Dr. Schürmann (Charité, Campus Virchow-Klinikum, Berlin, Germany).

### 4.3. Protein de-Glycosylation

Protein deglycosylation was performed with Peptide-N-Glycosidase F (PNGase F) according to the protocol of the supplier (New England Biolabs, Inc., Ipswich, MA, USA), using a cytoplasmic fraction of TZM-bl cells enriched with p48 KYNU.

### 4.4. SDS-PAGE, Native PAGE, and Western Blot Analyses

Electrophoresis was performed in a gradient of 4–20% SDS-PAG using Tris-Glycine buffer (Novex, Life Technologies, Carlsbad, CA, USA). Native PAGE was performed in 4–20% gels using Tris-Glycine native running buffer and SDS-free gel loading buffer (Novex, Life Technologies, Carlsbad, CA, USA). Gels were calibrated using PageRuler Plus pre-stained protein ladder and MagicMark XP Protein Standard ( ThermoFisher Scientific, Waltham, MA, USA) or Western blot standard (Serva, Heidelberg, Germany). Proteins were transferred onto 0.2 µm membrane (Protran BA83, Whatman GmbH, Dassel, Germany) at 45 V for 2 h. Membranes were blocked with 6% skimmed milk in PBS with 0.1% Tween 20 (blocking buffer) for 2 h at room temperature or overnight at 4 °C. Incubation with mAbs diluted in blocking buffer (1 µg/mL) was performed for 2 h at room temperature. After five times washing (5 min each) in PBS with 0.1% Tween 20 (PBS-Tween) membranes were incubated for 1 h 30 min with anti-human IgG-HRP conjugate (Dako Laboratories, Glostrup, Denmark) diluted 1:10 K in blocking buffer. The membranes were washed with PBS-Tween as indicated above, treated with Pierce ECL Western blotting substrate (Pierce, Rockford, Tempe, AZ, USA) for 1 min, and exposed to Amersham Hyperfilm ECL (GE Healthcare Limited, Pollards Wood, UK).

### 4.5. Confocal Microscopy

TZM-bl and HEK293T cells were seeded on poly-L-lysine coated glass-bottom µ-Dishes (Ibidi GmbH, Martinsreid, Germany). 24 h later the cells were washed with PBS and fixed with 2.5% paraformaldehyde for 10 min. The cells were washed twice with PBS, permeabilized for 10 min with 0.5% Triton X-100, washed three times with PBS, and blocked for 1 h with blocking buffer (1× PBS, 1% glycine, 0.1% Triton X-100) at room temperature. Then the cells were incubated for 1 h with 2F5 (10 µg/mL) diluted in blocking buffer, then rinsed three times in PBS with 0.1% Triton X-100 and stained with anti-human Alexa Fluor 488 conjugates (Invitrogen) diluted 1:200 in blocking buffer. Cells were left for 1 h at room temperature in the dark, rinsed three times with blocking buffer, and stained with DAPI as proposed by the manufacturer (NucBlue, Molecular Probes, Eugene, OR, USA). Images were captured using an LSM 780 fluorescent microscope (Carl Zeiss, Oberkochen, Germany) with an ×40 oil emersion lens. The LSM 5 Image Examiner software was used.

### 4.6. Isolation of p48 from Cell-Free Supernatant of TZM-bl Cells

Supernatants from TZM-bl cells grown on complete DMEM or SFM were harvested after 48 h, centrifuged at 1500 *g* and 10,000 *g* (each time for 10 min) to eliminate cells and large cellular debris, respectively. The protein from SFM was concentrated using MWCO 30 kDa filters (Sartorius Stedim Biotech, Germany). In addition, p48 was isolated by ultra-centrifugation of cell-free supernatant that was centrifuged at 170,000 *g* for 18 h at 4 °C. The pellets were suspended in PBS and were kept frozen at −80 °C before use.

### 4.7. Extraction of RNA and DNA

RNA was extracted using an RNeasy mini kit and RNase-free DNase set as recommended by the supplier (Qiagen GmbH, Hilden, Germany). The RNA specimens were immediately used in reverse transcriptase reaction. DNA was extracted from 2 × 10^6^ cells using a Blood and Tissue DNA isolation kit (Qiagen GmbH, Hilden, Germany). Quantification of nucleic acids was performed using the NanoDrop spectrometer ND-1000.

### 4.8. Reverse Transcriptase (RT)-PCR, Cloning, and Duplex Real-Time PCR

The RT-PCR was performed using Titan one tube RT-PCR system (Roche Diagnostics, Mannheim, Germany) and primers described in Table 2. The RT reaction was carried out at 46 °C for 40 min, followed by denaturation at 94 °C for 2 min and 35 cycles of amplification with 1 min of elongation steps, as recommended by the supplier. Amplicons were purified from agarose gel using Invisorb Spin DNA Extraction kit (Stratec Molecular GmbH, Berlin, Germany), cloned into pCR4-TOPO (Invitrogen Life Technologies, Carlsbad, CA, USA), and sequenced. For the protein expression, the amplicons were cloned into the pTargeT mammalian expression vector (Promega Corp., Madison, WI, USA). Transformed JM109 cells (Promega, Corp., Madison, WI, USA) were seeded on LB/ampicillin plates and kept for 16 h at 37 °C. The plasmids were isolated using the PureYield plasmid mini preps system (Promega, Madison, WI, USA) and analyzed. Sequenced was performed using BigDye terminator kit V.3.1. (Applied Biosystems, Foster City, CA, USA). The selected clone further used for transfection was called “pTargeT KYNU”.

The HIV-1 proviruses in Jurkat cells were quantified by real-time PCR (primers shown in Table 2) using the HIV-1 *gag* gene as a target sequence and glyceraldehyde-3-phosphate dehydrogenase (GAPDH) gene for normalization (ΔCt = Ct gene of interest − Ct GAPDH). Quantitative PCR was performed in duplicates in 25 µL (final volume) using SensiFast no ROX kit (Bioline GmbH, Luckenwalde, Germany). The thermal cycling profile was the following: enzyme activation at 95 °C-3 min and 42 cycles (95 °C/15 s-56 °C/30 s-72 °C/40 s), and a final hold at 4 °C. Fluorescence was measured using an Mx3005P Multiplex Quantitative PCR System (Stratagene, La Jolla, CA, USA).

### 4.9. Transfection

Transfection of the HEK293T cells (5 × 10^5^) was performed in 6 well plates, using 2 µg of pTargeT KYNU and 8 µL of TransIT-293 transfection reagent (Mirus Bio LLC, Madison WI, USA) per well. 48 h later the cells were harvested, washed three times in PBS, lysed on ice for 10 min using NP-40 cell lysis buffer, and centrifuged at 10,000× *g* for 10 min. The supernatant was harvested and used immediately in Western blot analysis, or it was kept frozen at −80 °C before use.

### 4.10. HIV-1_pNL4-3_ for the Neutralization (NT) Assay

The HIV-1 for the NT assay was obtained by transient transfection of HEK293T cells with infectious molecular clone pHIV-1pNL4-3 (2 µg of plasmid per 1 × 10^6^ cells) using TransIT-293 transfected reagent (Mirus Bio LLC, Madison, WI, USA). 48 h post-transfection the supernatant was harvested, centrifuged at 1500× *g* (10 min) and 10,000× *g* (10 min), filtered through the 0.45-µm filter, and stored at −80 °C before use. The virus titer was estimated on TZM-bl cells in triplicates using serial ten-fold dilutions. An aliquot of the supernatant was ultra-centrifuged at 170,000× *g* (SW41, Beckman, Pasadena, CA, USA) through a 20% sucrose cushion for 2 h. The viral precipitate was diluted in PBS to gain 200 times concentrate. The preparations were tested for virus integrity by Western blot using anti-p24CA (capsid), anti-gp41TM, and anti-gp120SU, as described [9]. Virus infectivity was estimated on TZM-bl cells using 96-well plates and supernatants from two independent transfection experiments. Briefly, 10 μL of the virus-containing supernatants from transfected cells were diluted in 90 μL of complete medium, titrated by serial ten-fold dilutions, and added to the TZM-bl cells. After 24 h the medium was replaced. 24 h later cells were washed twice with PBS and fixed with 2.5% paraformaldehyde for 5 min at room temperature. Fixed cells were washed with PBS and stained with X-gal (0.5 mg/mL) in PBS with 5 mM potassium ferricyanide, 5 mM Potassium ferrocyanide, and 2 mM MgCl_2_. The reaction was developed in the dark for 3 h at 37 °C or overnight at room temperature. Blue-stained (β-Gal+) cells were counted using a light microscope. Groups of blue-stained cells were counted as single foci of infection and wells containing >5 blue-stained cells were used for calculation.

### 4.11. NT Assay on TZM-bl and Jurkat Cells

TZM-bl cells were seeded in 96-well flat-bottom plates. The NT assay was performed the next day when the monolayer was ~80% confluent. The reaction mixture contained 10 µL (5 µg/mL or 20 µg/mL) of mAb 2F5, 5 µL of normal human serum, and different amounts of p48 KYNU (or rKYNU). The mixture was adjusted to 50 µL with complete DMEM (TZM-bl cells) or RPMI 1640 (Jurkat cells). HIV-1_NL4-3_ (initial infectious titer 2 × 10^5^/mL) was diluted in complete DMEM to gain the MOI 0.5 and added to each reaction mixture. Incubation was performed for 1 h at 37 °C and afterwards, the mixtures (100 µL) were loaded on TZM-bl cells. After 48 h cells were washed twice with PBS and fixed with 2.5% paraformaldehyde for 15 min and washed in PBS. The luminescence was measured using the Bright-Glo™ Luciferase Assay System (Promega, Madison, WI, USA) and the signal was estimated using GloMax^®^-96 microplate luminometer (Promega, Mannheim, Germany). The NT assay on Jurkat cells was performed as described above, but 2 × 10^5^ cells were used for infection, and dilutions were prepared using a complete RPMI 1640 medium. 48 h post-infection the Jurkat cells were harvested and washed twice with PBS. Extraction of DNA was performed using Blood and Cell Culture DNA Mini kit (Qiagen GmbH, Hilden, Germany). Proviruses were quantified by qPCR using the HIV-1 *gag* detection system.

### 4.12. Software

The Blast program (NCBI) was used for database search. Parameters of the oligonucleotides were examined using the OligoAnalyzer 3.1 (Integrated DNA Technologies, Coralville, IA, USA). The sequence alignments were performed using the software package Lasergene Version 10 (DNASTAR Inc., Madison, WI, USA). SecretomeP^2^ server (https://services.healthtech.dtu.dk/service.php?SecretomeP-2.0, accessed on 18 June 2016) was used to predict the possible way of KYNU secretion and UniProt resources (https://www.uniprot.org/, accessed on 19 June 2016) were used for the structural analyses. ENCODE (https://www.encodeproject.org/experiments/ENCSR329MHM/, accessed on 19 June 2016) transcriptome analysis was used to estimate the KYNU expression level in HepG2, HeLa, U87MG, and A549 cells. Octopus topology program (Stockholm University, Stockholm Bioinformatics Center, Stockholm, Sweden) was used to estimate transmembrane topology.

### 4.13. Statistics

The experiments with statistical evaluation were performed at least in triplicates. An unpaired t-test was used to evaluate the statistical significance. The *p* values less than 0.05 were regarded as statistically significant. Asterisks indicate: * *p* < 0.05; ** *p* < 0.01; *** *p* < 0.001; **** *p* < 0.0001. *p*-value calculations were performed using GraphPad Prism version 8.4.3. (GraphPad Software, San Diego, CA, USA; RRID: SCR_002798) software.

## 5. Conclusions

Here we demonstrated a vulnerability of the frequently used TZM-bl reporter cell line. The *L*-kynurenine hydrolase (KYNU), which contained the ELDKWA epitope recognized by mAb 2F5 was detected in these cells and cell supernatant.

- The amount of p48 KYNU in TZM-bl cells is nearly 0.64 pg/cell
- About 0.19 pg/cell of p48 KYNU are released to cell supernatant after 48 h
- From 7.5 ng to 9.35 ng of p48 KYNU can be released after 48 h from cells in one well of 96 well plate at the confluence.
- Released p48 KYNU can interact/inactivate 21–26 ng of 2F5-like antibodies in one well of 96 well plate.

Thus, 2F5-like antibodies in the TZM-bl NT assay can be partially or completely “inactivated” as a result of interference with the ELDKWA epitope of extracellular KYNU. The level of decrease depends on the proportion of neutralizing antibodies in the pool to antibodies recognizing the ELDKWA epitope. Consequently, the HIV-1 neutralization potency of previously tested sera reacting with gp41TM ELDKWA epitope might be underestimated.

Binding 2F5-like antibodies that were previously shown to recognize the ELDKWA epitope and demonstrated a so-called “week neutralization” of HIV in TZM-bl cell assay might contain significantly more neutralizing antibodies. In this regard revision of previously tested “weak” anti-ELDKWA sera might be essential and can shed light on the already designed immunogen that can induce 2F5-like antibodies. 

## Figures and Tables

**Table 1:** Cell lines.

Cell Lines	Origin	Cell Type/Morphology
HeLa	NIH AIDS Reagent Program (#153)	Human cervical carcinoma
HeLa CD4+LTR-Luc	Robert Koch Institute	Human cervical carcinoma
TZM-bl (HeLa-derived)	NIH AIDS Reagent Program (#8129)	Human cervical carcinoma
Huh 7.5.1.	Scripps Res. Inst., San Diego, CA, USA (Prof. Frank Chisari)	Human hepatocytes, epithelial-like
HepG2	ATCC, HB8065	Human hepatocytes
Jurkat	NIH AIDS Reagent Program (#177)	Human lymphocytes
Hepa 1-6	ATCC, CRL-1830	Mouse hepatoma
Vero	NIH AIDS Reagent Program (CCL-81)	Grivet monkey, kidney

**Table 2:** Primers and probes.

Name	Sequence 5′-3′	Nt. Position	Acc. Numbers
HIV env F1 (7531)	5′-CAGTGGACAAATTAGATGTTCATC	7531–7554	AF324493
HIV env F2 (7580)	5′-AGAGATGGTGGTAATAACAAC	7580–7600	AF324493
HIV env R1 (8475)	5′-GGCTCCGCAGATCGTCCCAGATAAGTG	8501–8475	AF324493
HIV env R2 (8347)	5′-TCCCTGCCTAACTCTATTCACT	8347–8326	AF324493
HIV *gag* F	5′-ATGGGTGCGAGAGCGTCGGTATT	790–811	AF324493
HIV *gag* R	5′-GGCTTCCTTGGTGTCTTTTACA	1089–1068	AF324493
HIV *gag* (Probe)	FAM 5′-AATCCTGGCCTTTTAGAGACATCAG-BHQ	928–950	AF324493
Kynu F	5′-ATGGAGCCTTCATCTCTTGAG	131–151	NM_003937
Kynu R	5′-TGCTCCTGCATTTAAATA	976–959	NM_003937
GAPDH F	5′-CCACTCCTCCACCTTTGAC-3’	1058–1076	NM_002046.5
GAPDH R	5’-ACCCTGTTGCTGTAGCCA-3′	1159–1142	NM_002046.5
GAPDH (Probe)	FAM 5′-TTGCCCTCAACGACCACTTTGTC-BHQ	1090–1112	NM_002046.5

## Data Availability

Data is available upon reasonable request.

## Supplementary Materials

The following supporting information can be downloaded at https://www.mdpi.com/article/10.3390/ijms23020641/s1.
