# Supplementary material for: The TZM-bl Reporter Cell Line Expresses Kynureninase That Can Neutralize 2F5-like Antibodies in the HIV-1 Neutralization Assay
Source: Int J Mol Sci. 2022 Jan 7;23(2):641. doi: 10.3390/ijms23020641 (PMC8775840; doi:10.3390/ijms23020641)
Supplement: Supplementary file 1 [file ijms-23-00641-s001.zip › ijms-1521977-supplementary.pdf]

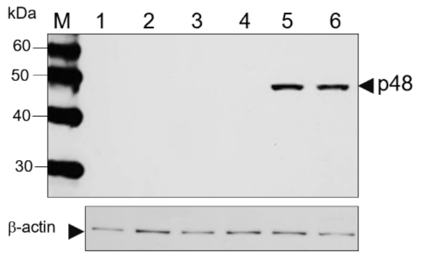

Figure S1. Expression of p48 in TZM-bl cells is independent of cell passage number. The whole-cell lysates from established cell lines were tested by Western blot analysis using mAb 2F5. M - ECL markers (Magic Mark XP Western blot standard, ThermoFisher Scientific). Tracks: 1- HEK293T cells; 2 - Jurkat cells; 3 - HeLa cells; 4 - Huh 7.5.1. cells; 5 and 6 -TZM-bl cells passages #10 and #25, respectively. Lower panel. Blot treated with mAb anti-β-actin.

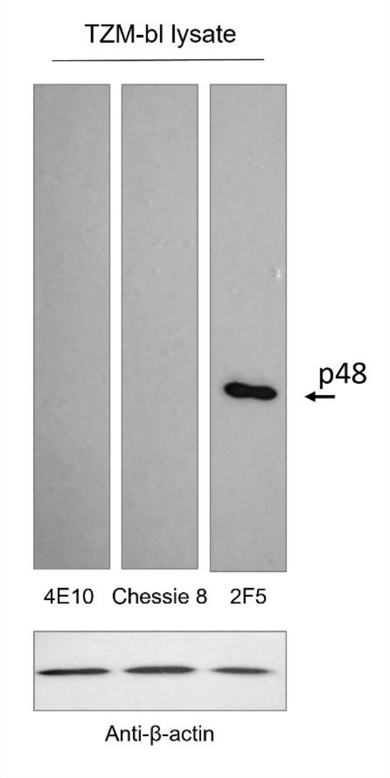

Figure S2. Lack of reactivity of 4E10 and Chessie 8 with TZM-bl cell lysate. The whole-cell lysates from TZM-bl cells were tested by Western blot analysis using mAb 2F5, 4E10, and Chessie 8. Lower panel. Blot treated with mAb anti-β-actin.

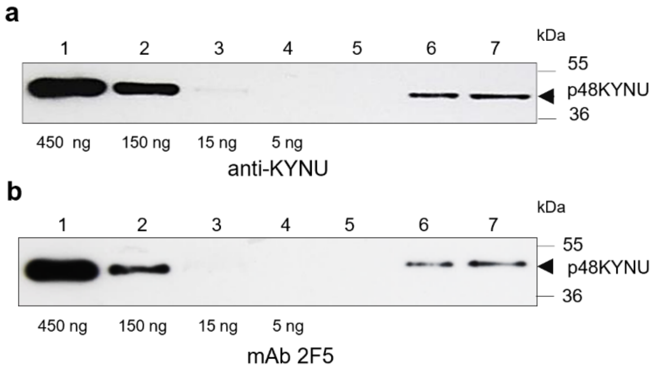

Figure S3. Comparative analysis of anti-KYNU and mAb 2F5 reactivity against rKYNU and p48KYNU. Reactivity of polyclonal anti-KYNU serum (a) and mAb 2F5 (b) with recombinant KYNU and p48KYNU from TZM-bl cells. Tracks: 1 - 450 ng of rKYNU; 2 - 150 ng; 3 - 15ng; 4 - 5 ng; 5 - 293T cell lysate from $4x10^4$ cells; 6 - TZM-bl cell lysate from $4x10^4$ cells; 7 - TZM-bl cell lysate from $8x10^4$ cells. Arrowhead indicates the position of KYNU. Rabbit anti-KYNU was used in dilution 1:750 and mAb 2F5 were diluted to 2,5 mg/ml. PageRulerTM Plus protein ladder (ThermoFisher Scientific) was used for gel calibration.

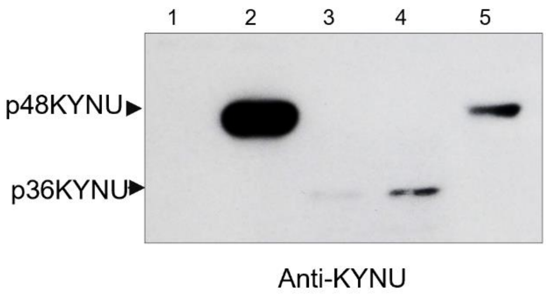

Figure S4. Detection of truncated recombinant KYNU (p36KYNU) in the lysate of transfected HEK293T cells. Tracks in order: 1- Lysate of HEK293T cells ($8x10^4$) transfected with empty vector; 2- Lysate of TZM-bl ($8x10^4$); 3- Lysate of transfected HEK293T cells ($4x10^4$); 4- Lysate of transfected HEK293T cells ($8x10^4$); 5- Lysate of HepG2 cells ($8x10^4$). Western blot analysis was performed using rabbit anti-KYNU serum (1:800).

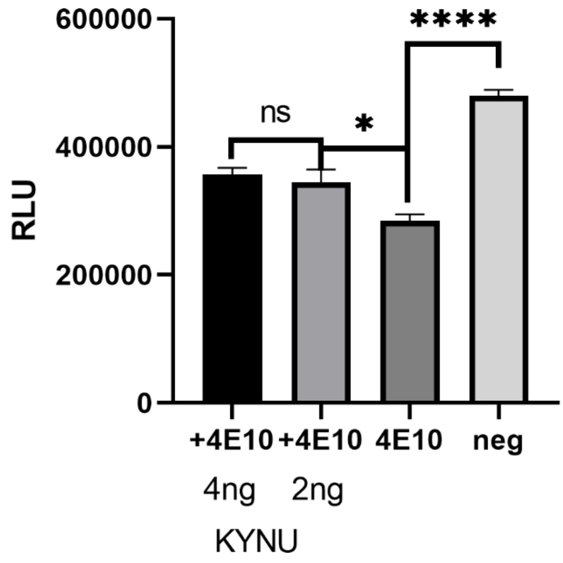

Figure S5. P48 KYNU has limited effect on mAb 4E10 in NT assay. The implication of p48KYNU on 4E10-like antibodies in NT assay. Amounts of p48KYNU added to the reaction mixture are shown below the columns. 4 ng and 2 ng of KYNU - with mAb 4E10 (indicated "+4E10"), respectively; 4E10- without additional KYNU. Neg. - negative control, reaction mixture without 4E10, or additional KYNU**.**
